# Hepatocellular carcinoma combined with sarcomatoid hepatocellular carcinoma: A case report

**DOI:** 10.1097/MD.0000000000037013

**Published:** 2024-01-26

**Authors:** Jingyi Li, Xizhuang Gao, Kun Zhao, Xiangzheng Meng, Shuwei Liu, Jian Zhang

**Affiliations:** a Clinical Medical College of Jining Medical University, Jining Medical University, Jining, Shandong, P.R. China; b Jining No.1 People's Hospital, Department of Radiology, Jining, Shandong, P.R. China; c Jining No.1 People's Hospital, Jining Medical University, Jining, Shandong, P.R. China.

**Keywords:** hepatocellular carcinoma, sarcomatoid carcinoma, sarcomatoid hepatocellular carcinoma, simultaneous occurrence

## Abstract

**Rationale:**

Sarcomatoid hepatocellular carcinoma (SHC) is an uncommon variant of hepatocellular carcinoma (HCC), characterized by HCC features combined with sarcomatoid histology and manifestations. The simultaneous occurrence of HCC and hepatosarcomatoid carcinoma is infrequent. This report presents a distinctive instance of HCC coexisting with hepatic sarcomatoid carcinoma in a 56-year-old male. The case exhibits an unusual clinical presentation, diagnosis, treatment, and prognosis. Through the presentation of this case, we aspire to contribute novel concepts to shape forthcoming strategies encompassing SHC diagnosis and treatment.

**Patient concerns:**

The 56-year-old male patient was admitted to the hospital, due to discovering a hepatic mass lasting for over 2 months.

**Diagnoses:**

Ultimately, combined hepatocellular and SHC diagnosis was conclusively confirmed through histopathological and imaging examinations.

**Intervention:**

In this case, our approach encompassed hepatectomy coupled with ultrasound-guided radiofrequency ablation for HCC. Intraoperative ultrasound localization was employed for accurate tumor identification, followed by postoperative hepatic artery embolization to facilitate meticulous tumor resection.

**Outcomes:**

He underwent hepatic arteriography chemoembolization treatment and is currently stable, experiencing regular chemotherapy follow-up visits.

**Lessons:**

The presence of distinct tumor types concurrently can influence treatment choices and prognosis. Given the intricate nature of this condition, crafting an optimal treatment strategy necessitates the incorporation of variables such as the patient age, tumor characteristics, liver function, and other pertinent factors.

## 1. Introduction

Sarcomatoid hepatocellular carcinoma (SHC) represents an infrequent subtype of hepatocellular carcinoma (HCC) exhibiting characteristics akin to HCC yet displaying concurrent sarcomatoid histology and clinical manifestations. Pathological features of hepatic sarcomatoid carcinoma encompass HCC cells exhibiting heterogeneous sarcomatoid proliferation. The tumor shows highly malignant attributes, including nuclear division, cellular anisotropy, and interstitial fibrosis. Hepatosarcomatoid carcinoma typically exhibits a more aggressive and malignant behavior than conventional HCC. Clinical presentations of hepatic sarcomatoid carcinoma resemble those of HCC, encompassing symptoms like liver mass, abdominal pain, fatigue, reduced appetite, and weight loss. Diagnosis is typically established through imaging modalities (e.g., ultrasound, computed tomography scan, magnetic resonance imaging [MRI], etc) and histopathological analysis (e.g., biopsy). Given the markedly malignant attributes of hepatosarcomatoid carcinoma, treatment poses challenges. Conventional therapeutic approaches encompass surgical resection, liver transplantation, local ablative therapy, chemotherapy, and targeted therapy.

Nevertheless, hepatic sarcomatoid carcinoma is associated with an unfavorable prognosis, characterized by a heightened incidence of postoperative recurrence and an overall dim prognosis. Our comprehension of SHC tumors and their epidemiology and associated risk factors remains limited, and a comprehensive diagnostic and treatment strategy has yet to be formulated. This study presents a case of concurrent HCC and hepatic sarcomatoid carcinoma. We comprehensively assess clinical characteristics, laboratory analyses, imaging findings, pathological presentations, treatment strategies, and predictive outcomes by reviewing pertinent national and international literature thoroughly.

## 2. Case presentation

### 2.1. General information

The 56-year-old male patient was admitted to the hospital on May 7, 2022, due to discovering a hepatic mass lasting for over 2 months. A middle-aged male with an unremarkable medical history, diagnosed with diabetes mellitus for 15 years. His highest fasting blood glucose level reached 28.8 mmol/L, typically managed with oral metformin and glibenclamide tablets. Additionally, he has a history of bilateral femoral head necrosis spanning over 20 years and thoracic hemangioma for 8 years. Over a decade ago, he underwent surgery at a local hospital to address a traumatic injury to his right knee. The procedure included an intraoperative blood transfusion.

Additionally, he has a history of significant trauma and experienced a traumatic cerebral hemorrhage 29 years ago, managed conservatively. During a magnetic resonance examination conducted at our hospital 2 months ago, numerous abnormal signals and enlarged retroperitoneal lymph nodes were observed in the liver. The possibility of a metastatic tumor was considered, leading to the performance of a hepatic puncture biopsy. The pathology examination revealed hepatocellular steatosis with increased infiltration of inflammatory cells, focal hepatocellular atypical hyperplasia, and immunohistochemistry indicated the disappearance of small bile ducts with hepatic sinusoids undergoing capillarization; the possibility of HCC was not ruled out. Immunohistochemistry results showed positive staining for hepatocyte and CK8/18, negative staining for Glypican-3, positive staining for CD10 in capillary bile ducts, positive staining for CK19 in small bile ducts, positive staining for CD34 in vasculature, a few positive staining for GS, negative staining for SMA, and about 3% positive staining for Ki-67 (Table 1). Upon admission, the examination revealed no jaundice of the skin and sclera, a flat abdomen, absence of gastrointestinal patterns or peristaltic waves, abdominal softness, lack of pressure or rebound pain, absence of palpable liver and spleen beneath the ribs, no abdominal masses, a negative Murphys’ sign, no percussion pain in the liver and kidney regions, and absence of mobile turbidities. The blood test results are presented in Table 2.

**Table 1:** Tumor pathological classification and immunohistochemical indexes of the patient.

Components	CK	CK7	CK8/18	CK19	Hepatocyte	GS	AFP	Glypican-3	Ki-76
SHC	+	+	+	+	−	+	−	−	+40%
HCC	+	−	+	−	+	+	−	+	+25%

AFP = alpha-fetoprotein, SHC = sarcomatoid hepatocellular carcinoma, HCC = hepatocellular carcinoma.

**Table 2:** Laboratory test results of the hepatocellular carcinoma combined with hepatic sarcomatoid carcinoma.

Items	Factors	Values	Reference range
Tumor markers	AFP (ng/mL)	10.30	0–7.02
	CA-199 (U/mL)	71.40	0–27
Liver function tests	AST (U/L)	23.4	15–40
	ALT (U/L)	22.5	9–50
	TBIL (U/mL)	14.3	7–23
	DBIL (U/mL)	6.3	0–10.3
	IBIL (U/mL)	8.0	0–15.2

AFP = alpha-fetoprotein, ALT = alanine aminotransferase, AST = aspartate aminotransferase, T-Bil = total bilirubin, D-bil = direct bilirubin, I-BIL = indirect bilirubin, CA19-9 = carbohydrate antigen 19-9.

### 2.2. Treatment and surgical procedures

Bite hardness, distinct boundaries, and incomplete encapsulation. The more significant lesion in segment VII measured approximately 3.7 × 3.5 × 2.0 cm and was near the right hepatic vein and the inferior vena cava. Liver segment III measured 3.1 × 2.7 × 2.0 cm and was contiguous with the absent section of the left portal vein branch. Through repeated ultrasonographic assessment, a superficial hepatic tumor of approximately 1.0 × 1.0 × 0.5 cm was identified in segment V. Multiple enlarged lymph nodes were evident within the abdominal cavity and adhesions of the gastrointestinal and stomach walls. One month after surgery, liver function remained within acceptable limits. Transcatheter arterial embolization was done by puncturing the right femoral artery under local anesthesia using the Seldinger technique. A catheter was then inserted, with a preference for abdominal trunk insertion, followed by a digital subtraction angiography examination. Small areas of anomalous staining were observed in the liver parenchyma, supplied by a branch of the proper hepatic artery. Once the arterial branching in the hepatic region was confirmed through microcatheter superselection, delivered via the catheter, embolization was achieved by simultaneously injecting a mixture of 3 mL of iodinated oil and a suspension of Pirarubicin 30 mg through the microcatheter under fluoroscopic guidance. After the procedure was completed, digital subtraction angiography was conducted, resulting in the resolution of the abnormal staining. Iodized oil deposits became evident, and vascular casts were observed. The microcatheter was removed. Then, the catheter was retracted to the peritoneal trunk to administer 100 mg of oxaliplatin and 20 mg of Pirarubicin through the catheter. Finally, the catheter was removed upon completion of the procedure. Regular postoperative treatment included targeted therapy with Lenvatinib (Levima) and injectable pembrolizumab immunotherapy.

### 2.3. Post-operative pathology

**Figure 1.**
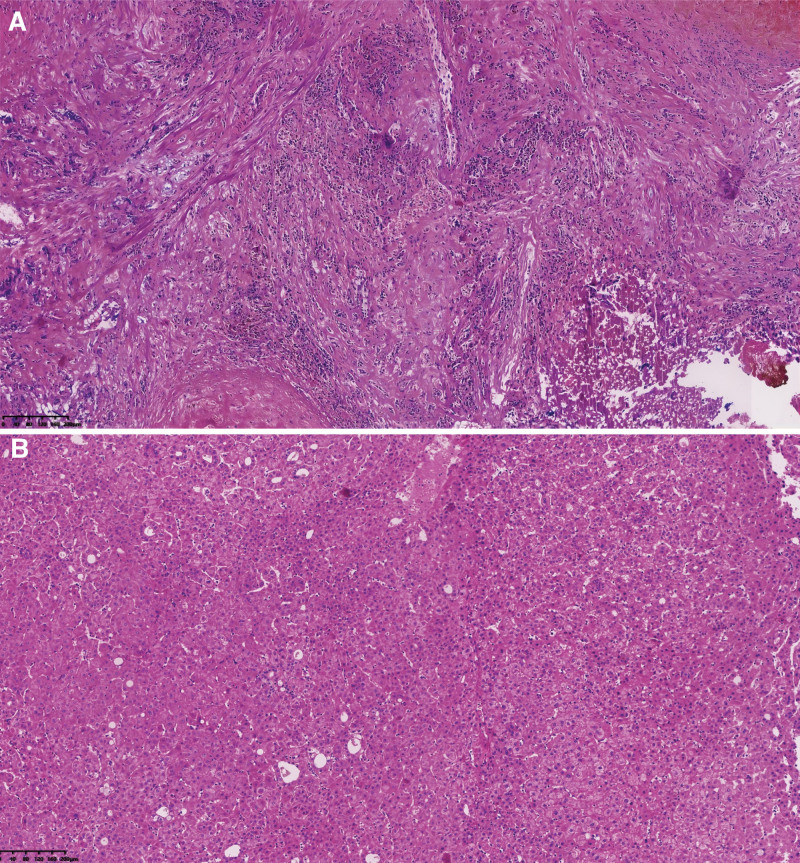
Displays HE-stained sections of the tumor group after surgical intervention. (A) Sarcomatoid histiocytes exhibit disorganization, characterized by large nuclei with intense staining, abundant cytoplasm, prominent nuclear anisotropy, and visible spindle cells (×100); (B) normal lobules exhibited disruption, with fibrous septa, disorganized hepatocytes, and slightly dark-stained, mildly heterogeneous nuclei (×100). HE = hematoxylin and eosin.

**Figure 2.**
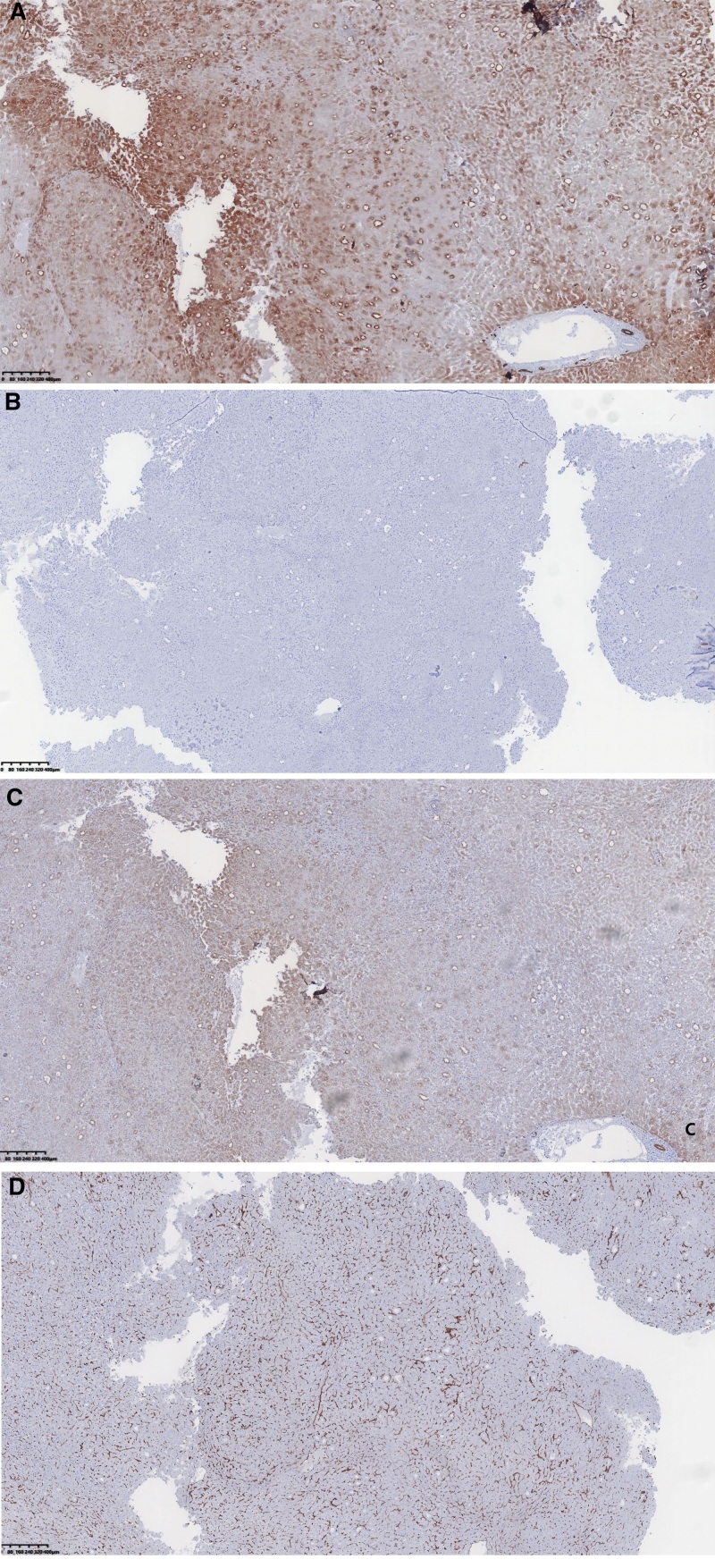
Illustrates the immunohistochemical staining outcomes for sarcomatoid carcinoma in liver segment VII. (A) CK(+) (×100); (B) CK19(+) (×100); (C) CK8/18(+) (×100); (D) vimentin(+) (×100).

**Figure 3.**
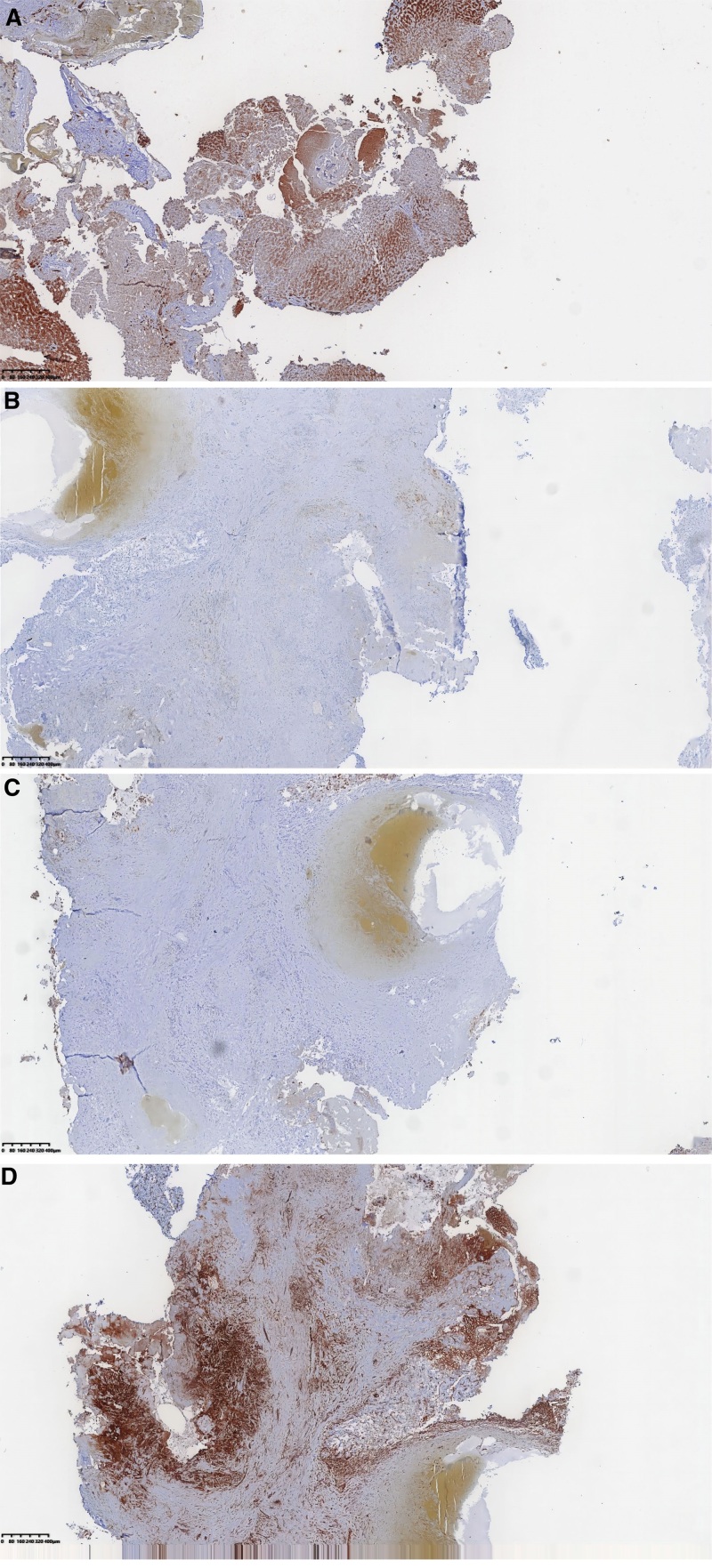
Displays the immunohistochemical staining outcomes for hepatocellular carcinoma in liver segment VII. (A) CK(+) (×100); (B) CK19(+) (×100); (C) CK8/18(+) (×100); (D) vimentin(+) (×100).

**Figure 4.**
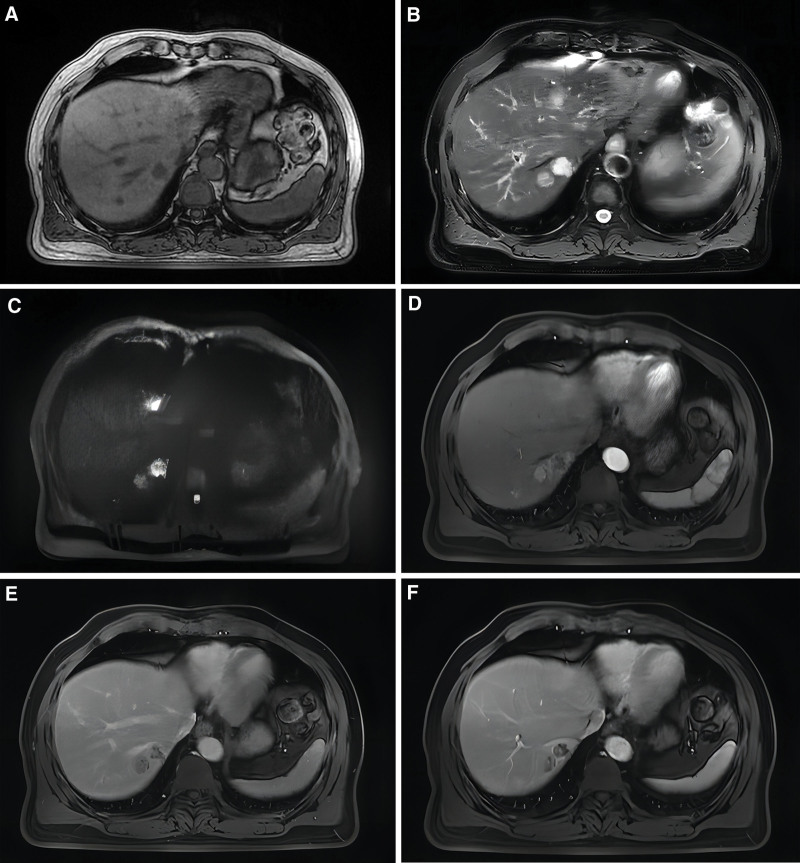
Abdominal MR image before surgery. (A) Lesion shows a mild low signal on T1WI; (B) soft downward motion on T2WI; (C) heterogeneous high signal on DWI sequence; (D) patchy enhancement is seen around the lesion in the arterial phase; (E) low signal in the delayed phase; (F) everyday movement in the portal phase.

**Figure 5.**
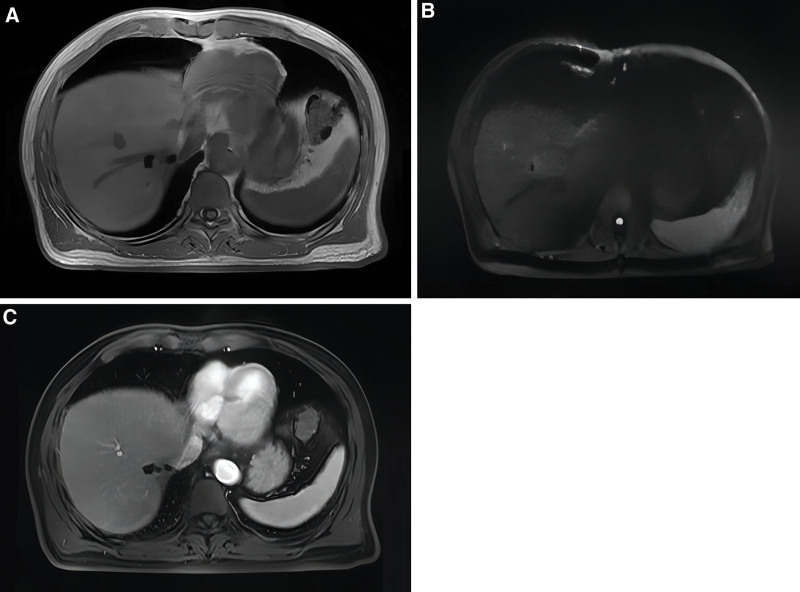
Postoperative MR image of the abdomen. (A) Numerous areas with patchy prolonged T1 signal shadows are observed in the surgical site. (B) A patchy high DWI signal is detected at the periphery of the surgical site in the left liver lobe. (C) The enhanced scan reveals nonsignificant enhancement.

**Figure 6.**
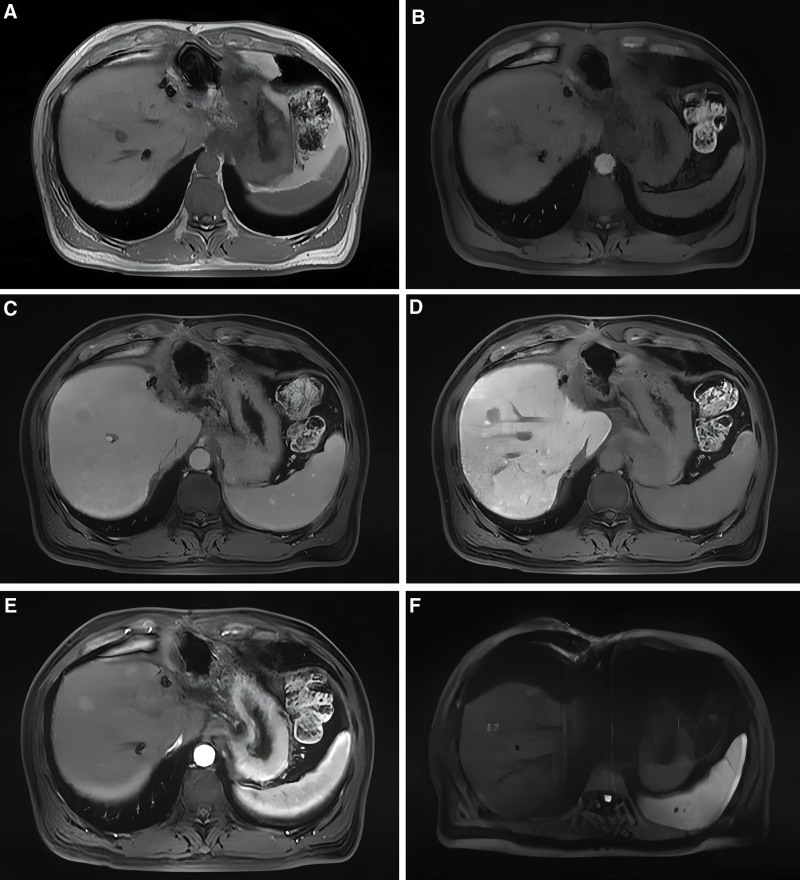
New hepatocellular carcinoma in segment SVII was detected during a 5-mo postoperative abdominal MRI follow-up. (A) A slightly shorter rounded T1 signal shadow is observed in the hepatic segment SVII; (B) a round enhancing shadow is visible in the arterial phase; (C) enhancement diminishes in the delayed phase; (D) low signal in the hepatobiliary phase; (E) enhancement decreases in the portal venous phase; (F) no prominent abnormally restricted high-signal shadow is observed in DWI. MRI = magnetic resonance imaging.

Macroscopic observation revealed a liver segment V tumor measuring approximately 5.5 × 4.5 × 2 cm. The cross-section displayed shades of grayish-red and grayish-brown, with a medium texture and a ligamentous structure. Liver segment VII tumors: Small tumor measuring approximately 2 × 2 × 0.5 cm, and large tumor measuring about 3.7 × 3.5 × 2 cm. The giant tumor appeared grayish-white on the section and exhibited brittleness. Hematoxylin and eosin staining reveals the presence of a large tumor in hepatic segment VII. The hepatic sarcomatoid histiocytes display a disorganized arrangement characterized by substantial cytoplasm, large nuclei with intense staining, and prominent nuclear heterogeneity. A small tumor was observed in liver segment VII, described by disrupting the typical lobular structure, visible fibrous septa, disorganized arrangement of hepatocytes, slightly dark-stained nuclei in specific cells, and mild anisotropy (Fig. 1). Immunohistochemical findings show that A sizable tumor is located in liver segment VII (Fig. 2). The tumor cells show positive staining for CK8/18, CK7, CK19, GS, CK, EMA, CAM5.2, and vimentin; however, they are harmful to alpha-fetoprotein (AFP), Glypican-3, HMB45, SMA, MealanA, and hepatocyte (Fig. 3). The proliferation marker Ki-67 is positively expressed, with approximately 40% of cells exhibiting staining. Small tumors are observed in hepatic segment VII, characterized by positive staining for CK8/18, Glypican-3, GS, CK, hepatocyte, and Ki-67 while showing negative staining for AFP, CK7, and CK19. The proliferation marker Ki-67 is positively expressed, with approximately 25% of cells exhibiting staining. Pathological Diagnosis: 1. Large tumor located in liver segment VII: Poorly differentiated carcinoma, suggestive of sarcomatoid carcinoma. 2. Small tumor in hepatic segment VII: Moderately differentiated HCC with nodular cirrhotic changes in the adjacent liver tissues. In addition, we added, pre-surgical, post-surgical and 3-month post-surgical follow-up MR images of the abdomen (Figs. 4–6).

### 2.4. Diagnoses

HCC (T4N1M0, stage IVA), SHC (T2N1M0, stage IVA), abdominal lymph node metastasis, portal vein tumor embolism, hepatic damage, intestinal adhesions, pleural effusion, peritoneal effusion, varicose veins of the lower extremities, old cerebral hemorrhage, type 2 diabetes mellitus.

### 2.5. Prognosis

A follow-up abdominal MRI was conducted after 5 months, revealing a novel abnormal signal in liver segment VII. This newly identified signal was indicative of a recurrence of HCC. Numerous aberrant signals are detected in the liver, suggesting the possibility of cirrhotic regenerative nodules, hepatic atypical hyperplastic nodules, and multiple small retroperitoneal lymph nodes. Tumor biomarkers were assessed: AFP level at 11.77 ng/mL, CA-199 level at 51.50 U/mL, and CEA level at 4.25 ng/mL. He underwent hepatic arteriography chemoembolization treatment and is currently stable, experiencing regular chemotherapy follow-up visits.

## 3. Discussion

HCC and SHC represent distinct tumor entities within the liver. While their concurrent manifestation is uncommon from a clinical standpoint, the rarity challenges physicians and pathologists. This unusual simultaneous presence could arise due to many intricate elements, including genetic mutations, the tumor microenvironment, and liver development.

### 3.1. Potential mechanisms

The concurrent development of HCC and hepatosarcomatoid carcinoma lacks a definitive explanation. One hypothesis suggests that while these 2 tumors might arise from distinct liver cell types, they could still be influenced by shared oncogenic factors. For instance, long-term hepatitis virus infection, cirrhosis, and fatty liver potentially contribute to the development of both types of tumors.^[1]^ An alternative hypothesis posits that HCC and hepatosarcomatoid carcinoma could share common precursor lesions. These lesions might serve as the initial stage for the concurrent development of both tumor types. Numerous prior studies have demonstrated that individuals diagnosed with HCC may exhibit a sarcomatoid transformation following diverse anticancer interventions such as transarterial chemoembolization, radiofrequency ablation, or percutaneous ethanol injections.^[2,3]^ Nevertheless, the current case emerged without any prior recurring interventions, rendering it an even rarer occurrence. Consequently, we posit that the etiological underpinnings of SHC encompass a broader array of factors than HCC.

### 3.2. Diagnostic challenges

Clinical and imaging manifestations of HCC and hepatosarcomatoid carcinoma may inevitably overlap, rendering the concurrent presentation more challenging to diagnose. HCC typically presents distinct cytological characteristics and dynamically enhanced computed tomography or MRI images. Conversely, hepatic sarcomatoid carcinoma often demonstrates an undifferentiated embryonic appearance. This similarity contributes to the potential misdiagnosis of the already infrequent SHC as a liver abscess in clinical settings.^[4]^ In this instance, the additional MRI of the patient revealed a slightly prolonged T1 and T2 signal on scanning. Patchy enhancement surrounding the lesion was observed during the arterial phase, while no prominent display was evident during the portal venous phase. These findings align with the imaging characteristics documented in earlier case reports.^[5,6]^ The clinical presentation of these 2 tumors can exhibit notable similarities. SHC, in particular, often presents with fever, chills, abdominal pain, and weight loss.^[7]^ Consequently, this also gives rise to diagnostic challenges. Thus, the definitive diagnosis of SHC relies heavily on accurately employing histopathological, immunohistochemical, and pathological methodologies. In contrast to HCC, hepatic sarcomatoid carcinoma exhibits reduced differentiation and a more irregular cellular morphology. Hepatosarcomatoid carcinoma is distinguished by pronounced cellular heterogeneity, where cells exhibit non-uniformity in size and shape. Furthermore, nuclei within the carcinoma may display morphological heterogeneity.^[8]^ While immunohistochemical profiling has provided specific diagnostic insights into SHC, the identification of disease-specific biomarkers remains elusive up to the present. SHC lesions exhibit positivity for both epithelial markers (cytokeratin) and mesenchymal markers (vimentin).^[2]^ In this particular case, the patient exhibited positivity for CK8/18, CK19, CK, and Vimentin, along with a notably elevated Ki-67 index. The pathological attributes of the lesion closely aligned with findings from earlier investigations of SHC.^[9,10]^ Furthermore, these observations could potentially offer valuable points of reference for subsequent cases.

### 3.3. Treatment

Surgical resection stands as the foremost practical approach for SHC treatment. Nonetheless, it noteworthy that post-surgical local recurrence and distant metastasis occurrences tend to be more prevalent in SHC patients than those with classical HCC. Consequently, the aggressive nature of SHC surpasses that of conventional HCC, subsequently leading to an inferior prognosis.^[11,12]^ In this case, our approach encompassed hepatectomy coupled with ultrasound-guided radiofrequency ablation for HCC. Intraoperative ultrasound localization was employed for accurate tumor identification, followed by postoperative hepatic artery embolization to facilitate meticulous tumor resection. Consistent with prior case reports, the patient encountered a recurrence 5 months post-surgery. Nonetheless, the patient current condition reflects stable vital signs.

## 4. Conclusion

To summarize, we present an uncommon instance of concurrent HCC and hepatic sarcomatoid carcinoma. Furthermore, it is worth noting that conventional liver tumor interventions, including surgery, chemotherapy, and radiotherapy, might yield restricted efficacy in SHC management. Moreover, hepatic sarcomatoid carcinoma typically demonstrates a highly aggressive growth pattern, potentially facilitating swift infiltration into neighboring tissues and organs, thereby engendering the development of locally advanced pathology. This heightened aggressiveness often culminates in the creation of tumors that pose substantial challenges to surgical excision or the attainment of complete resection. In this instance, following surgical intervention, even though the patient vital signs have stabilized, imaging continues to indicate indications of recurrence, Consequently, an immediate requirement exists for an increased case pool to facilitate advanced research endeavors to investigate more efficacious therapeutic approaches, ultimately enhancing patient survival and quality of life. Moreover, through the presentation of this case, we aspire to contribute novel concepts to shape forthcoming strategies encompassing SHC diagnosis and treatment.

## Acknowledgments

We would like to extend our heartfelt gratitude to the individuals and organizations who have contributed to the completion of this research study. This work would not have been possible without their support, expertise, and resources. We want to acknowledge the contributions of our research team members, Jingyi Li, Xizhuang Gao, Kun Zhao, Jian Zhang, Xiangzheng Meng, and Shuwei Liu, for their dedication and collaboration in various aspects of this project, including data collection, analysis, and manuscript preparation. Despite the lack of financial support, heartfelt appreciation is extended for the significant contributions made by the authors mentioned above.

## Author contributions

**Investigation:** Kun Zhao, Xiangzheng Meng, Shuwei Liu.

**Writing – original draft:** Jingyi Li.

**Writing – review & editing:** Xizhuang Gao, Jian Zhang.
